# Unbusinesslike Nurses

**Published:** 1909-05-01

**Authors:** 


### Nursing Administration.

### UNBUSINESSLIKE NURSES.

The accounts which have been coming in relating to the working of the Midwives Act throw a good deal of light on the habits of nurses in respect of notification. The process of conforming to the law in the case of midwives is a dual one. They must first be registered once for all by the Central Midwives Board; they must next notify the local supervising authority of their intention to practise as midwives, and this is a form which has to be gone through every year. Now, although every woman in *bona fide* practice as a midwife was for some years admitted to the Roll of Midwives without examination, a very large number of practising midwives neglected even the simple formality of getting their name inscribed. They ignored the provisions of the Act altogether, and have gone on practising ever since as though it had never been framed. It is quite impossible to estimate the number of these uncertified women, but if all were included who take occasional cases it is probable that the number would run into some thousands. Another large class of midwives take the initial step, inscribe their names on the roll, and omit to notify to the supervising authority. These women are almost as much outside the reach of the Act as the others. They hinder its proper working, and in spite of all the efforts of hardworking inspectors, their work remains uninspected, and is only brought into prominence in the event of an inquest. The report of the Central Midwives Board states " it does not seem extravagant to estimate that the number of certified midwives in practice at the present time cannot fall far short of 15,000." Of these, during 1907, only 12,964 had given the required notice to the supervising authorities of their intention to practise. In other words, a seventh part of the whole body of certified midwives had omitted to take the trouble to fill in a form of notification and pay the required fee of 2s. 6d., for the purpose of making their position legally secure. In particular counties the proportion of the unnotified is far higher; as, for instance, in Middlesex, where it is stated there are 299 notified midwives and 469 midwives who have not notified. In the face of these facts what probability is there of bringing all the trained nurses in the country into line as members of a recognised body, duly inscribed in a central roll, with their names duly verified year by year? The Central Midwives Board has the following aids towards securing registration and notification:—

1. A heavy penalty is attached to failure on the part of the midwife to register or notify.
2. Any birth at which she officiates must be duly registered by an independent authority.
3. Her duties are performed under rules which render it necessary for medical aid to be summoned under various well-defined conditions, and it is therefore easily possible to place the midwife under inspection.
4. The registration and notification fees are low.
5. The number of midwives in the kingdom is comparatively small, and their qualifications are confined to one special branch of knowledge.

If under these comparatively easy conditions it has hitherto been impracticable to ensure complete registration and notification, how will any Nursing Registration Board cope with a problem in every particular more complex?

1. There will be no penalties for nurses who do not register or notify.
2. The ordinary duties of a nurse are performed in strict privacy.
3. No inspection is possible, because the inspector cannot penetrate to the bedside of the nurse's patients, nor can the nurse be compelled to summon medical aid under special conditions, except in the case of infectious disease.
4. The fee suggested for registration and notification is largely in excess of that required from midwives.
5. The vast numbers and variety in qualification of trained nurses introduce features of special difficulty in the task of keeping them in touch with a supervising authority.

The controversies surrounding the question of registration are slowly shifting from the question, " Shall nurses be registered? " to the more complex problem, " How shall nurses be registered? " This being the case, it is all important that one great factor in the whole matter, the unbusinesslike qualities of nurses, shall be allowed full weight. Medical men who take up the subject of registration are apt to overlook this exceedingly important aspect of the registration problem. They argue from their own experience. Who ever heard of a doctor in good practice neglecting to secure that his name was on the register? The members of Parliament who throw themselves generously into the cause of nurse registration cannot conceive of a profession containing thousands of women who rather than take the trouble to pay a yearly half-crown, will deprive themselves of the right to a State qualification. But matrons who have daily evidence of the casual ways of the most highly-skilled nurses in things which belong to their interests are not hopeful of united action on the part of the nursing profession unless the act of registration can be settled for the candidate as an integral part of certification by the training school. Unless this is done, unless, as in the recent proposals for a rational system of registration, the examination is held by the various hospitals, and the certificates are sent up for registration to the central body so that no trained nurse is ever allowed to go out into practice unregistered, there must inevitably result two bodies of trained nurses, the one registered and the other unregistered, for the confusion of the public, and the stultification of a costly State system. It will undoubtedly be a great step gained to get all trained nurses entered on a central roll. That they can ever be induced with or without penalties to verify their names every year for the purpose of keeping the roll correctly, is shown by the action of the certified midwives, a large proportion of whom are trained nurses, to be altogether outside the bounds of probability.

